# Nurse-Led Virtual Delivery of PIECES in Canadian Long-Term Care Homes to Support the Care of Older Adults Experiencing Responsive Behaviors During COVID-19: Qualitative Descriptive Study

**DOI:** 10.2196/42731

**Published:** 2022-12-13

**Authors:** Anna Garnett, Denise Connelly, Marie-Lee Yous, Lillian Hung, Nancy Snobelen, Melissa Hay, Cherie Furlan-Craievich, Shannon Snelgrove, Melissa Babcock, Jacqueline Ripley, Pam Hamilton, Cathy Sturdy-Smith, Maureen O’Connell

**Affiliations:** 1 Arthur Labatt Family School of Nursing Western University London, ON Canada; 2 School of Physical Therapy Western University London, ON Canada; 3 School of Nursing McMaster University Hamilton, ON Canada; 4 School of Nursing University of British Columbia Vancouver, BC Canada; 5 Registered Practical Nurses Association of Ontario (WeRPN) Mississauga, ON Canada; 6 Health and Rehabilitation Sciences Western University London, ON Canada; 7 Vision 74 Inc Sarnia, ON Canada; 8 Copper Terrace Long-term Care Home Chatham, ON Canada; 9 Pieces Canada Kingston, ON Canada; 10 Pieces Canada Owen Sound, ON Canada; 11 Pieces Canada Barrie, ON Canada

**Keywords:** long-term care, older adults, families, responsive behaviors, qualitative, COVID-19, PIECES, nurse(s), care home, infection, therapeutic, anxiety, depression, cognitive, fear, death, dementia, communication, technology

## Abstract

**Background:**

Worldwide, the COVID-19 pandemic has resulted in profound loss of life among older adults living in long-term care (LTC) homes. As a pandemic response, LTC homes enforced infection control processes, including isolating older adults in their rooms, canceling therapeutic programs, and restricting family member visits. Social isolation negatively impacts older adults in LTC, which may result in increased rates of anxiety, depression, physical and cognitive decline, disorientation, fear, apathy, and premature death. Isolation of older adults can also cause an increase in responsive behaviors (eg, yelling, hitting, calling out) to express frustration, fear, restricted movement, and boredom. To respond to the challenges in LTC and support frontline staff, older adults, and family members, a novel registered practical nurse (RPN)-led delivery of the PIECES approach for addressing responsive behaviors among older adults with dementia using virtual training/mentoring was implemented in Canadian LTC homes. PIECES employs a person- and family/care partner–centered collaborative team-based approach to provide education and capacity-building for nurses; engages families as active participants in care; and embeds evidence-informed practices to provide person- and family-centered care to older adults with complex needs, including dementia.

**Objective:**

The aim of this study was to describe the experiences of LTC staff, family/care partners, and older adult research partners with implementation of a novel RPN-led virtual adaptation of the PIECES care-planning approach for responsive behaviors in two Canadian LTC homes during the COVID-19 pandemic.

**Methods:**

Using a qualitative descriptive design, two focus groups were held with three to four staff members (eg, RPNs, managers) per LTC home in Ontario. A third focus group was held with three PIECES mentors. Individual semistructured interviews were conducted with RPN champions, family/care partners, and older adult research partners. Research team meeting notes provided an additional source of data. Content analysis was performed.

**Results:**

A total of 22 participants took part in a focus group (n=11) or an in-depth individual interview (n=11). Participant experiences suggest that implementation of RPN-led virtual PIECES fostered individualized care, included family as partners in care, increased interdisciplinary collaboration, and improved staff practices. However, virtual PIECES, as delivered, lacked opportunities for family member feedback on older adult outcomes. Implementation facilitators included the provision of mentorship and leadership at all levels of implementation and suitable technological infrastructure. Barriers were related to availability and use of virtual communication technology (family members) and older adults became upset due to lack of comprehension during virtual care conferences.

**Conclusions:**

These findings offer promising support to adopting virtual PIECES, a team approach to gather valuable family input and engagement to address residents’ unmet needs and responsive behaviors in LTC. Future research should investigate a hybridized communication format to foster sustainable person- and family-centered care-planning practices to include active collaboration of families in individualized care plans.

## Introduction

Globally, the COVID-19 pandemic has resulted in death and decline in the physical, mental, and behavioral health of older adults in long-term care (LTC) [1,2]. This population has been more vulnerable to the COVID-19 virus than the general population in part due to their living situation, advanced age, less robust immune system, and increased likelihood of having multiple comorbidities [3]. In Canada, two-thirds of COVID-19 deaths were among older adults in LTC from March 11, 2020, to May 8, 2020 [1]. In the early stages of the pandemic, LTC homes prioritized containing the spread of COVID-19 through strict infection control processes, which included isolating older adults in their own rooms, pausing therapeutic activities, and imposing restrictions on visits by family members. These measures meant that many older adults experienced prolonged periods of seclusion and a loss of social engagement. The social isolation of older adults in LTC homes was further exacerbated by government-mandated restrictions on in-person gatherings, which compounded the already stringent restrictions in place across many LTC homes [4].

Outcomes of social isolation among older adults include a heightened risk of experiencing anxiety, depression, physical and cognitive decline, and premature death [5-7]. Other impacts include worsening apathy and boredom [8,9]. Many older adults’ family members provide up to 30% of care, including assistance with personal care, nutrition, as well as social stimulation and emotional support [10]. These family members are also recognized by the LTC home staff as key partners in the care of older adults; however, current practice models used to guide care planning and delivery do not readily include them as active team members [11]. As a result, prohibiting caregivers from in-person visits with older adults in LTC in the earliest waves of the pandemic served to exacerbate older adults’ social isolation and the subsequent health and behavioral consequences.

Prior to the pandemic, 50% of older adults with dementia in LTC experienced responsive behaviors (eg, yelling, restlessness, hitting) in response to unmet needs such as pain, boredom, thirst, and hunger [12]. Multiple factors within LTC contribute to disproportionate responsive behaviors, including lack of staff education, inadequate skills, lack of support from leaders in management, and insufficient staffing resources [13]. Isolation of older adults with dementia can lead to higher incidences of responsive behaviors with a resultant increased use of antipsychotic medications by LTC staff [14]. During multiple waves of COVID-19, increased incidence of responsive behaviors in older adults in LTC was documented with the concomitant widespread isolation resulting from infection control processes and restrictions on in-person gatherings [15,16]. Approximately 51% of family members have reported an increase in responsive behaviors in persons with dementia since the pandemic started [15]. This exacerbation in behaviors highlights the need for interventions to support older adults and the care staff, and calls attention to the need to engage all persons caring for them (eg, older adults, families, frontline staff) in care-planning processes (eg, team-based) and policy development (eg, infection control and family as essential care partners, not visitors).

An example of a team-based care-planning model is PIECES, which consists of a holistic, relationship-based, and evidence-informed team approach to collaborative assessment and shared care designed for use by interdisciplinary teams to address the complex physical and mental health (eg, cognitive, emotional, social, psychological) needs of older adults [17]. Understanding what matters to the person and family is central to the PIECES model. The PIECES approach includes the following components in assessing the individual: Physical, Intellectual, and Emotional health; maximizing the Capabilities of an individual to support quality of life; integrating the living Environment of a person; and encompassing a person’s Self, including beliefs, culture, and life story [17]. PIECES has been implemented across Canada by interdisciplinary teams and in diverse settings, including acute care, LTC, home and community care, complex continuing care, and mental health settings, to put a plan in place to address responsive behaviors.

The PIECES approach educates staff to recognize and address mental health challenges and responsive behaviors, leading to more thorough assessments using a variety of existing tools, strengthening partnerships between LTC homes and external resources, and improving family satisfaction with care delivery [18,19]. In acute care settings, PIECES enhances collaboration between different disciplines, promotes individual assessments of older adults, and facilitates problem-solving to address behaviors [19,20]. By virtue of its person-centered design, the PIECES approach is well-suited to be delivered by registered practical nurses (RPNs), as the largest regulated frontline staff in LTC homes [21] who are most familiar with the older adults’ day-to-day behavioral expressions and changes. In-person delivery of PIECES has been demonstrated to improve the recognition and early management of responsive behaviors and communication between staff and family; however, it has not previously been implemented virtually [20]. Virtual care is well-placed to support interdisciplinary team-based care planning by enhancing team communication, providing continuity of care, and facilitating collaborations between specialists such as geriatricians, physicians, and mental health experts [22-24]. Moreover, virtual care may offer novel training and leadership opportunities for frontline RPNs to lead care discussions involving multiple parties (eg, family care partners, health care providers, older adult LTC residents) regardless of their physical location.

During the COVID-19 pandemic, an urgent need emerged for increased support for older adults experiencing responsive behaviors in LTC [22]. This study employed a virtual adaptation of PIECES [17] training using Personal Health Information Protection Act–approved Zoom videoconferencing technology. Synchronous meetings using virtual technology for RPNs to conduct individualized family care conferences with the family off-site, the older adult, and other health care team members (on- or off-site) were used to create an integrated care plan. This virtual PIECES intervention, shepherded by an on-site RPN champion from within the partner LTC home, provided the health care team with care protocols, supported by behavioral support staff, to maintain quality in care practices during the COVID-19 pandemic and inclusion of family in care decisions.

PIECES-trained staff completed PIECES referrals for residents experiencing responsive behaviors with the collaboration of other staff members (eg, personal support workers, social workers, recreational therapists, nurses) in the LTC home. RPN champions then contacted family members to schedule a virtual family care conference to discuss the behaviors of residents and to put in place a plan of care to address these behaviors. The RPN champion, PIECES-trained staff, family member, and resident (if able and willing) were present at the care conferences held by Zoom. PIECES-trained staff or RPN champions followed up with families to provide telephone or in-person updates regarding the outcomes of strategies put in place to address responsive behaviors. The role of PIECES mentors was to provide PIECES training for LTC staff, debrief with PIECES-trained staff and RPN champions, and address any questions. Understanding the facilitators and barriers to virtual PIECES implementation is an important future step to sustain and spread virtual care delivery. The purpose of this study was therefore to describe experiences and implementation facilitators and barriers in delivering a novel RPN-led virtual adaptation of the PIECES care-planning approach to address behavioral expressions in two Canadian LTC homes during the COVID-19 pandemic.

## Methods

### Study Design

This study used a qualitative descriptive design, which allows for the provision of a detailed description and low-inference interpretations of data, defined as remaining close to the words of participants [25]. The Consolidated Criteria for Reporting Qualitative Research (COREQ) statement was used as a reporting guideline (see Multimedia Appendix 1) [26].

### Setting

Two LTC homes in Ontario, Canada, were involved to inform and host implementation of a virtual PIECES intervention. There was no pre-established relationship between the research team and the LTC homes prior to the study. Both sites are considered as midsized privately owned LTC homes (138 and 146 beds, respectively) that have been in operation for more than 30 years; one home operates as a for-profit venture and the second as nonprofit, offering varying services (eg, family physician, nursing and personal care, housekeeping, meals, laundry, leisure activity programming, nutritional services, and services within the community such as physiotherapist, social worker, X-ray, and ultrasound services).

### Sample and Recruitment

Study participants consisted of RPN champions, PIECES mentors, PIECES-trained LTC staff of various disciplines (eg, nursing, nutrition, recreation, behavioral management), family/care partners, and older adult research partners. The older adult research partners who participated in this study were selected based on the clinical judgment of the director of care in the LTC home. The director of care has a nursing background and purposely selected the two residents to be research partners, as they were able to understand the purpose of the study and contribute through virtual means. Convenience sampling [27] was used to select these participants. The participants were key stakeholders in implementing virtual PIECES at the LTC homes and family/care partners, who were active members of virtual care conferences to provide the family/resident story and to discuss mitigating the responsive behaviors of older adults. Participants were invited to engage via email or by phone. Participants were able to read, understand, and speak English. The sample size was not predetermined. However, review of qualitative descriptive studies showed that most studies included 11 to 25 participants [28]. The sample size was deemed sufficient once diverse data were obtained and overlapping data indicated the approximation of data saturation [29,30].

### Data Collection

Study processes were explained to all participants who then provided written consent for their involvement in the study. All focus groups and interviews were conducted using Zoom videoconferencing by two female postdoctoral fellows with experience in qualitative research (MLY, MH). A total of three focus groups were held. Two focus groups were held with three to four PIECES-trained staff of each LTC home (ie, one focus group per LTC home). A focus group was also held with the three PIECES mentors. Since the PIECES mentors regularly engaged with the LTC staff members (eg, behavioral management lead, registered dietitian, RPNs, managers) throughout the implementation of virtual PIECES, their perspective was important to inform understanding of the facilitators and barriers to this process. These focus groups were 60-80 minutes in length. Individual semistructured interviews were conducted with RPN champions, family/care partners, and older adult research partners. These interviews lasted between 30 and 70 minutes, and no repeat interviews were conducted. Focus group and interview guides (see Multimedia Appendix 2 for sample questions) were developed through a review of the literature on various concepts, including PIECES, virtual care conferences, responsive behaviors, older adults, and LTC, and feedback of the research team members. Participants were asked about their experiences with the virtual PIECES intervention, implementation facilitators and barriers, and recommendations for improvement of the intervention. Interviews were recorded, anonymized, and transcribed verbatim. Field notes were documented by two interviewers with training in qualitative data collection methods (MLY, MH) throughout meetings and interviews.

### Data Analysis

Qualitative content analysis was used to summarize data and form interpretation of key messages as well as provide rich, detailed descriptions of the entire data set [25]. This inductive analytic method allows for identifying, describing, interpreting, and summarizing emerging patterns within the data while ensuring themes are linked to the voices of participants [31-33]. Open coding was used to develop themes, which offered a general description of the experience [34]. Analysis of interview transcripts, as well as observational field notes, allowed the research team to explore organizational factors, implementation process, team engagement, experiences in using technology, and the virtual care planning from the perspectives of key stakeholders. Preliminary codes and themes were generated by two research team members (MLY, AG) and then consensus of thematic inclusion was reached with all research team members. Data analysis included reflexive participant collaboration wherein RPN champions, PIECES mentors, LTC staff, and older adult research partners provided ongoing feedback on interpretations to support participant validation in the presenting of findings [35,36].

### Rigor and Trustworthiness

Approaches were used to uphold rigor and trustworthiness in qualitative research, including addressing Lincoln and Guba’s [37] criteria: credibility, transferability, dependability, and confirmability. Investigator triangulation of research team members with experience supporting older adults and family/care partners in LTC, addressing responsive behaviors, and qualitative research ensured credibility of the findings. This strategy was also found to complement and support the validation of data [37]. Comprehensive and detailed descriptions of the setting and sample of the study were used to ensure that the findings were transferable [37]. The research team ensured that processes of the study were informed logically by conducting a comprehensive review of the existing literature to determine gaps in the literature.

### Ethical Considerations

Ethics approval was granted from the local University Ethics Boards (#118629 and #H21-01428). All participants received a written introduction to the study and an informed consent form written in lay language, which they signed prior to participating in the study. Study IDs were assigned to participants to maintain anonymity and participation was voluntary. The three core principles—respect for persons, concerns for welfare, and justice—of the Tri-Council Policy Statement [38] were enacted throughout the study. A Can $25 gift card (approximately US $20) was provided to all nonacademic research team participants in recognition of their study participation.

## Results

### Demographic Characteristics

A total of 22 participants took part in a focus group (n=11) or interview (n=11) during the midstage of virtual PIECES implementation. Most of the participants were female (90.9%). Both older adult research partners were male. They were involved in reviewing information and providing feedback on research outputs such as manuscripts and presentations. There was diverse representation of team members. LTC staff had various roles within the LTC homes (see Table 1). Limited demographic information was collected as not to burden participants in addition to the time they had already contributed to the study.

**Table 1:** Descriptive characteristics of the participants.

Variable		Participants, n
**Gender**		
	Female	20
	Male	2
**Role**		
	Family/care partner	7
	LTC^a^ staff (eg, behavioral management lead, registered dietitian, RPN^b^, manager)	8
	PIECES mentor (eg, physiotherapist, psychogeriatric resource consultant, manager of specialized geriatric services)	3
	RPN champion	2
	Older adult research partner	2

^a^LTC: long-term care.

^b^RPN: registered practical nurse.

### Themes

#### Overview of Themes

Resultant themes were grouped according to: (1) participant experiences, (2) implementation facilitators, and (3) implementation barriers of the virtual adaptation of the PIECES approach (see Textbox 1 for an overview of themes). The two LTC homes are identified as Site 1 and Site 2, respectively, and participant IDs are used when providing direct quotations.

**Overview of themes.**

**Experiences**

- Virtual PIECES provided individualized care for older adults
- Families recognized as partners in care through virtual PIECES
- Virtual PIECES led to greater interdisciplinary collaboration
- Virtual PIECES improved the practice of long-term care (LTC) staff
- Virtual PIECES contained limited opportunities for families to provide feedback on outcomes

**Implementation facilitators**

- Huddles were helpful for interdisciplinary team involvement
- Mentorship provided support and encouragement for staff to deliver virtual PIECES
- Leadership in all forms was key to implementing virtual PIECES
- Equipping LTC homes with technological infrastructure was essential

**Implementation barriers**

- Availability and use of virtual communication technology
- Concerns surrounding the presence and involvement of older adults at virtual care conferences

#### Experiences

##### Experiences Subthemes

Overall participating family/care partners, LTC staff, RPN champions, PIECES mentors, and older adult research partners reported positive experiences engaging with the virtual adaptation of the PIECES approach. Emergent themes of their experiences with virtual PIECES demonstrated that it: (1) provided opportunities for individualized care for older adults, (2) recognized families as partners in care, (3) led to greater interdisciplinary collaboration, (4) improved the practice of LTC staff (eg, communication with family), but (5) contained limited opportunities for families to provide feedback on outcomes.

##### Virtual PIECES Provided Individualized Care for Older Adults

Virtual PIECES was perceived by family/care partners, staff, older adult research partners, and PIECES mentors as a way to ensure that older adults were provided with individualized care to meet their needs and abilities. Family/care partners felt that, through virtual PIECES, staff were making greater efforts to relate to older adults on a more personal level.

I think it [Virtual Care Conference] was more personalized for mom because the annual meeting, it’s just more or less an update for me to let me know her weight and when they bathe her and what her diet is and how physiotherapy is going so that’s mainly what that annual meeting is about but this meeting a couple of Fridays ago was more on a personal level and an emotional level just trying to get in touch with mom as far as her personality goes rather than her physical care. So that was nice.

Site 1, FCP 2

PIECES conversations helped to build trust with older adults, which led to greater family satisfaction with care. Some family/care partners stated that they learned more about their family member through virtual care conferences, such as the occurrence of new behaviors and the severity of depression. Staff perceived that interactions with family/care partners and older adults were more positive through PIECES and they took more time to get to know older adults. Staff who participated in PIECES had knowledge regarding the older adults’ preferences that were shared with them by family members during care conferences. This enabled the staff to share the older adults’ preferences with other staff members so they could better meet the older adults’ needs on a consistent basis. These staff also tailored their care to optimize independence among older adults as much as possible. “Capabilities too, like if she’s [older adult] capable [to instill her own eye drops] and she can do it and she wants to stay as independent as possible that makes her feel glad that she can maintain that” [Site 1, Staff 1].

Videoconferencing used in delivering PIECES provided comfort for older adults, which enabled them to discuss their care with staff in the presence of a family/care partner.

So specifically with one of our PIECES referred resident, this resident did attend the care conference and she was delighted and eager to see her family. It was lovely to see their interactions so it was nice to kind of observe that interaction between the family member and the resident. We were able to discuss and involve that resident in her care and her actions so that was really nice to be able to involve the resident because that is in our bill of rights that each resident, they have the right to receive their own care and be involved in their own care plan.

Site 2, Staff 1

RPN champions noted that there was improved rapport between family care partners, older adults, and staff as a result of individualized care delivered using virtual PIECES.

##### Families Recognized as Partners in Care Through Virtual PIECES

In the implementation of virtual PIECES, families were formally recognized as care partners. Family/care partners appreciated that PIECES staff sought their input and were asking questions about their older adult family members. Families believed that their perspectives were being heard by staff and that staff implemented specific actions to address their feedback.

I just feel like any other conversations that we had regarding mom were kind of like dismissed or ignored and not even brought in the loop about things that were happening so I I think this has been huge that calls are made, that people are talking to me about it and saying this is what we’re seeing, what is your feedback? How can we help with that? How can we do this so that the staff isn’t frustrated, your mom’s not frustrated and we’re all working together, that makes sense to me but that’s not how it was so I feel like finally that’s what’s happening

Site 2, FCP 3

Prior to virtual PIECES, some family/care partners had limited interactions with LTC home staff unless an emergency arose. As a result of virtual PIECES, family/care partners felt they were welcome to call staff and ask questions about their family members. Staff and RPN champions similarly acknowledged the value of seeking input of families in care planning and to ensure that families’ concerns are being acted upon.

I mean family has always been part of the team and with our care conferences, our annual care conferences and family has always been encouraged to come in. I definitely think PIECES has enhanced that family portion and definitely has enlightened the family on the different lens that we are looking at from a health perspective.

Site 2, Staff 1

Using videoconferencing for care conferences allowed family members to participate in care planning regardless of their physical location.

##### Virtual PIECES Led to Greater Interdisciplinary Collaboration

Virtual PIECES enabled greater interdisciplinary collaborations to provide care for older adults in LTC: “I think that a multidisciplinary approach to our intervention gives us the best information that we can. It helps solve our investigation from all sorts of perspectives and viewpoints” [Site 2, Staff 1]. The success of the intervention was perceived by LTC staff, RPN champions, and PIECES mentors as involving various disciplines (eg, nursing, directors of care, recreational therapy, housekeeping).

Everybody, absolutely everybody is part of the team from the directors to the cleaning lady, the PSWs [personal support workers], the nurses, the RNs [registered nurses], physios, the kitchen staff, the laundry staff. Everybody is working in conjunction to make their home the best possible experience for them so I see everybody involved.

Site 1, FCP 2

Staff perceived that they were better able to work with different services such as Behavioural Supports Ontario to address responsive behaviors of older adults. At Site 2, the Geriatric Mental Health Outreach team was so impressed with the implementation of virtual PIECES that they reached out to staff to inquire about the referral processes used.

##### Virtual PIECES Improved the Practice of Staff

LTC staff, RPN champions, and PIECES mentors perceived that virtual PIECES encouraged more holistic assessments as routine practice in addressing responsive behaviors:

You get the chance to look at the person holistically and see what we’re missing and then the PIECES assessment helps you do that and it takes you through physical and emotional cognitive abilities. So then by going step by step, you can actually say okay, I’m missing this person or I’m missing this step, they’re not on the nursing rehab program, I’ve missed the pain component. I think it’s just highlighted it and making it actionable.

Site 2, RPN Champion 1

LTC staff had a better understanding of triggers, were less task-oriented, and better able to prioritize care. They became more confident in addressing behaviors and were now looking for inconsistencies in resident assessments. “We check for irregularities...dehydrated she could get confused and agitated because she’s not eating and not drinking. We addressed that because of PIECES” [Site 1, Staff 2]. Staff were more knowledgeable in seeking input from families and communicating with them. This helped fill a gap in LTC about how best to involve older adults and families in care. Staff experienced resilience in moving forward with PIECES despite COVID-19 challenges. The PIECES-trained staff team was seen by other staff members as experts.

##### Virtual PIECES Contained Limited Opportunities for Families to Provide Feedback on Outcomes

Although virtual PIECES involved family, there was a lack of feedback opportunities for family/care partners regarding what actions have been taken as a result of meetings. Family/care partners were interested in knowing whether suggestions were being used by staff and whether the older adults’ behaviors had improved.

All I would say is maybe more feedback or more detail of what is being done. We had no idea about, we didn’t know about virtual [PIECES] being used or what it is was or how it was used...All we got in the conference was that the social worker spends an hour a day with her, that’s all we got. What they do in that hour, I have no idea.

Site 2, FCP 5

One family/care partner suggested that a phone call would suffice to provide an update. One PIECES mentor similarly recognized the need to seek feedback from families on the implementation process of virtual PIECES.

The one thing I did want to mention is how critical it is in their organizations that they’ve gone back to their family, the families who have been part of this to kind of use as a reference check for their implementation process so I didn’t want to forget them because they have been a big part in the implementation in their respective organizations

PIECES Mentor 3

#### Implementation Facilitators

##### Implementation Facilitators Subthemes

Implementation facilitators of the virtual adaptation of the PIECES approach themes included: (1) huddles were helpful for interdisciplinary team involvement, (2) mentorship provided support and encouragement for staff to deliver virtual PIECES, (3) leadership in all forms was key to implementing virtual PIECES, and (4) equipping LTC homes with technological infrastructure was essential.

##### Huddles Were Helpful for Interdisciplinary Team Involvement

Huddles (ie, LTC staff unit-specific meetings) to engage the interdisciplinary team in virtual PIECES were formally implemented at Site 2. These were helpful in discussing older adults with new responsive behaviors to better understand their past history.

[RPN Champion] had one [huddle] the other day. We were just discussing a new resident and some care approaches too and just like background information on her so just to better understand how to properly care for her and manage her behaviors.

Site 2, Staff 2

The huddles were found to be a helpful strategy in relieving staff of the burden to seek individual input from various disciplines in their completion of assessments as all staff members were gathered in one meeting. This was especially important when staff were faced with limited time and heavy workloads.

Those huddles are definitely very beneficial. A great way to get information to front line staff very quickly to a mass audience so that huddle and identifying the short form, we really can review and complete a short form in a short amount of time with a multidisciplinary team. The short form essentially can be completed with the input from all the team members at a huddle and there’s multidisciplinary team members coming as well. There was dietary, management, front line, PSWs, RPNs, RNs.

Site 2, Staff 1

##### Mentorship Provided Support and Encouragement for Staff to Deliver Virtual PIECES

Virtual PIECES delivery was made possible through mentorship provided by managers, senior staff members, and PIECES mentors. PIECES mentors were found to be excellent support for staff as they were experts in PIECES, provided encouragement, and celebrated staff accomplishments.

I was hesitant. [PIECES mentor 2] was very helpful. She really got us through this and I told her now I’m writing a book of [PIECES mentor 2] like focus on her positives and priorities and so I have her now in the back of my mind and saying these things and I carry that out on the floor when I’m working and I thought no matter how fast the pace is, taking a few minutes of extra time, I think that’s really what we’re taking away from this and I think it’s a constant learning, I know more this week than last week and I think there are more residents in each Zoom meeting we’ve had

Site 1, Staff 1

PIECES mentors ensured that information was consistently delivered across homes. LTC home administrators were supportive of staff and RPN champions, and made certain that they were provided with dedicated time to participate in training of PIECES and complete assessment forms. A natural partnership occurred between Site 1 and 2 as a result of the virtual PIECES implementation, and both sites supported each other through sharing of resources such as financial report templates and algorithms that worked well for implementing virtual PIECES*.*

[Manager] has been involved and she’s done a lot of offering ideas and asking for what I would think if I was on the unit. She’s done a lot of the process planning and has said do you think that would work or not. And then you guys as well and also collaborating with [Site 2], it has been useful to hear their ideas and how they’re doing it. It has been very helpful. I think we’ve adapted to some of their ideas and maybe vice versa, I don’t know if they’ve used any of our ideas but their algorithm really helped get ours going because I think we had a hard time starting it.

Site 1, RPN Champion 1

##### Leadership in All Forms Was Key to Implementing Virtual PIECES

The key to implementing virtual PIECES was the support provided by various leaders such as managers and RPN champions. Staff perceived that RPN champions were excellent role models for the rest of staff. Some staff members also assumed the role of leaders in implementing virtual PIECES and led team huddles.

I know [Site 2 Staff 2]’s here for one of her resources shifts and she was doing that, formally huddling the staff and you could see that she was quite confident and knowledgeable and asking questions to kind of help her to understand what was happening.

Site 2, RPN Champion 1

Managers and administrators of LTC homes were very supportive of staff in implementing virtual PIECES and saw a need for the intervention due to the increase of responsive behaviors.

The support from leadership and they’ve had tremendous support and that needs to be on an ongoing way...What is going to be so essential is the ongoing support from leadership and a commitment and this is with all organizations and however big or large the team and whether along continual care, that leadership continues pave the way for the implementation in practice and there has certainly been tremendous leadership support that’s just so critical as we move forward

PIECES mentor 1

##### Equipping LTC Homes With Technological Infrastructure Was Essential

Staff were able to implement virtual PIECES with the support of their managers and administrators with regard to access to technology and training: “I was not very familiar with Zoom...We’ve come a long way from not knowing how to plug in the TV” [Site 1, Staff 1]. Videoconferencing was new for many older adults, families, and staff. Over time, and with the technological support and resources, families and staff have become more confident in using Zoom for meetings: “I don’t know whether it was a 1- or 3-month difference...so we are making headway with Zoom and I think people are becoming more used to the virtual communication” [Site 2, Staff 3]. Managers recognized the need to ensure that equipment is being stored securely, devices are connected, and clear instructions/resources are provided for staff and families in using technology: “[Manager] is really instrumental as to finding a place to store the technology, getting it in place, writing out the templates for the staff so I feel that was really well managed” [Site 2, RPN Champion 1].

#### Implementation Barriers

##### Implementation Barriers Subthemes

Despite the numerous facilitators and positive experiences with the virtual adaptation of the PIECES approach, there were some barriers reported, including: (1) availability and use of virtual communication technology, and (2) concerns surrounding the presence and involvement of older adults at virtual care conferences.

##### Availability and Use of Virtual Communication Technology

Virtual PIECES was generally well-received by older adults, family/care partners, and staff. Some family/care partners were comfortable in using virtual communication technology for work-related meetings and other events. Other family/care partners were unfamiliar with the technology.

I had to get help with my daughter, she helped set me up but I’m learning. I will learn eventually how to do it myself, it’s just going to take me awhile, I have to see it and do it a few times but I’m finally getting the hang of using my phone regularly, it’s an iPhone. I know how to make phone calls and I know how to go online but it’s hard.

Site 1, FCP 6

The study team offered tech support (ie, information technology [IT] squad for a driveway visit) to support family members, but this was declined by participating family members. Other participants recalled minor issues with technology such as connectivity challenges leading to frozen screens or no sound available during family care conference meetings.

Sometimes there’s technical difficulties so there could be no sound on one meeting and then another meeting we couldn’t get a picture so when things work out just so, you’ve really climbed a mountain and things are great so the challenge would be more so just the technical aspect of things.

Site 2, Staff 1

##### Concerns Surrounding the Presence and Involvement of Older Adults at Virtual Care Conferences

While staff and family/care partners appreciated the involvement of older adults in care planning, the extent of meaningful engagement at virtual care conferences may depend on individual circumstance. Staff and family/care partners perceived that advanced cognitive impairment among older adults may cause them to be upset during meetings as they may not be able to comprehend conversations.

Having the resident present, sometimes it’s harder though depending how advanced their dementia is, like sometimes it’s very upsetting to some residents but if they are able to understand what’s happening, it’s like an interaction so really, very helpful, that the volume is up and the screen is large and if you figure things out, it runs very smoothly...I think the challenges really happen when residents aren’t willing to stay because this has been upsetting to them, like we had one, and she said that is not my daughter, what have you done to my daughter, and it got to be too much, so we had to remove her.

Site 1, Staff 1

Some issues raised were of a sensitive nature, such as new behaviors, and this may have been upsetting for older adults to hear. The sensory impairments of older adults were also perceived by family/care partners as impacting their ability to participate in virtual care conferences due to decreased hearing and sight.

How do you get the nuances of a virtual call when you’re 80 and you’ve never done it and you can’t hear very well or your hearing’s not as great so I think for us it was probably easier to have the call with [Site 2 Staff 1] with mom not there to actually talk about the issues and I know it’s hard, I mean it is good to have mom’s perspective but I also do think that [Site 2 Staff 1] also sees her and talks to her too.

Site 2, FCP 3

## Discussion

### Principal Findings

To our knowledge, this is the first study to explore the experiences and implementation barriers and facilitators of a virtual adaptation of the PIECES approach in an LTC setting. Overall, virtual PIECES was well-received by participating family/care partners, LTC staff, PIECES mentors, RPN champions, and older adult research partners. Participants described how virtual PIECES supported care planning to address responsive behaviors and provided an opportunity to offer individualized care within the challenging context of the COVID-19 pandemic. Study findings suggest that virtual PIECES has value beyond the pandemic and is an important approach to engage family/care partners in LTC homes who may otherwise be excluded from care processes. LTC staff and family/care partners described how virtual PIECES helped them to provide care that was individually tailored to the life stories, preferences, and abilities of older adults.

Collaboration between family/care partners and LTC staff helped facilitate successful responses for older adults who were experiencing responsive behaviors. Prior research has shown that family members are well-positioned to support LTC staff in recognizing changes in an individual’s health because of their extensive familiarity with them [39]. Benefits of virtual care conferences, such as ease of access and inclusivity, were also appreciated by family/care partners and LTC staff. Family/care partners felt they could contribute important information to inform the older adult’s care and were also given opportunities to learn about care provision by the LTC home. Despite the well-known benefits of family involvement in care-planning processes in LTC, research conducted in the United States found that only 16% of family/care partners participated in care-planning processes [40]. Older adults with more advanced forms of cognitive impairment may require greater involvement of family in care planning as they are unable to verbally communicate their needs and wishes, yet half of older adults in LTC with severe cognitive impairment do not have a family/care partner involved in their assessments [40]. Steps to foster person- and family-centered care can involve collaborative discussions that engage family members through interprofessional education for LTC staff and through the provision of opportunities for staff and family to attend care conferences [41,42]. Interventions in LTC that involve partnerships between family and staff often lead to positive outcomes such as improved quality of life for older adults and decreased burden and depression for caregivers [43,44]. Furthermore, these successful interventions also encouraged multiple key stakeholders to help engage family in care planning [43].

In this study, participants perceived that virtual PIECES, with its emphasis on inclusive and regular engagement and communication, enhanced individualized care for older adults experiencing responsive behaviors. Family/care partners felt that LTC staff knew the older adults on a more personal level and engaged with genuine interest to improve care delivery. This helped to strengthen and build trusting relationships between LTC staff, families, and older adults. Older adults who were unable to take part in virtual care conferences were well-represented by family/care partners who advocated for them, discussed medications and potential triggers for responsive behaviors, and could represent the older adults in decision-making processes, which are all key components of person- and family-centered care [45]. Positive relationships between older adults, family, and staff, as well as regular communication are necessary for collaborative decision-making regarding care [46,47]. Moreover, older adults and/or their families should be engaged in the development of personalized care plans in LTC [40]. Findings from this study highlight the important role that LTC leadership has to facilitate different approaches to person- and family-centered care, a finding that is also documented in the research literature [41]. Virtual communication technology, as implemented in this study, is a recognized tool to support older adults’ social connections with their family members [16].

Our findings further demonstrated that the virtual care conferences and team huddles implemented as part of virtual PIECES facilitated the ability of family/care partners and LTC staff to develop care plans to address responsive behaviors. Virtual care conferences held either by video or teleconference have been found to reduce stress among families and providers and increase the confidence of staff in addressing responsive behaviors [48]. Research on a video-consultation program to link health experts with multidisciplinary LTC staff to develop care plans to address responsive behaviors resulted in new recommendations that were followed for 72.3% of older adults [49]. However, despite being invited to participate in care-based discussions to address older adults’ behaviors, families rarely participated [49]. This finding suggests there is a need for improved communication, education, and efforts to foster an inclusive environment so that families are familiar and comfortable with the meaningful role they can have at care conferences. Going forward, it will be important to evaluate the impact of interventions including virtual PIECES on resident outcomes in LTC, such as quality of life, responsive behaviors, agitation, pain, and depression, and fostering LTC staff and family/care partners relationships.

Ensuring that LTC homes had sufficient technological infrastructure was necessary for the successful implementation of virtual PIECES in this study. Family/care partners and LTC staff experienced challenges with the virtual communication technology at varying points, which included a lack of familiarity and receiving insufficient training to use the technology. Identified benefits of using technology to implement virtual PIECES included the ease of providing mentorship through virtual platforms and the facilitation of education to enhance staff practice. This research also highlights policy implications, including an identified need to provide support to families and staff in LTC to use technology, and ensuring that financial and human resources are available to offer technology as a mode of communication for all older adults and families during the pandemic and beyond. Integral to this process would be addressing aspects of equity; providing dedicated funding and IT resource personnel to assist families in accessing internet-based platforms. LTC homes should promote the use of technology among families by conducting online case conferences and family council meetings. Further, it will be important to ensure that diversity and inclusion are considered in the promotion and use of technology for family engagement. This may require closed captioning, an interpreter, language translation, and/or inclusion of culturally relevant care elements in LTC care models. Videoconferencing rather than telephone calls should be encouraged so that emotional connections and familiarity are nurtured by being able to recognize people on screen, and to see facial expressions and nonverbal cues [50]. They should ensure that homes have good quality WiFi in all locations—internal and external to the building—that staff receive technological training and support, and that LTC staff job descriptions recognize the role of staff to assist older adults and family/care partners to engage with technology [16]. Findings from this study suggest that family/care partners are interested in the outcomes of virtual conferences and value opportunities to provide feedback. Henceforth, family/care partners should have opportunities for structured and predictable communication where they can connect with both older adults and the LTC staff [50]. During and beyond pandemics and outbreaks, there would be a benefit to having key staff members assigned as primary contacts for family/care partners to facilitate ongoing tailored communication using video, telephone, or email [51].

### Strengths and Limitations

This study exhibits numerous strengths, including participants from key stakeholder groups representing multiple roles within the LTC home; participation of older adults and family/care partners in collaborating in research; in-depth exploration of experiences with the virtual care–planning process, including facilitators and barriers related to implementation of virtual PIECES; and the use of several strategies to ensure the trustworthiness and rigor of the study findings. Moreover, this is the first known study to implement a virtual adaptation of the PIECES approach in LTC. Limitations identified were the inclusion of only two LTC homes located in Ontario, Canada; the use of single interviews that may have constrained the ability to capture implementation experiences over time; and a lack of participant demographic information such as ethnicity, age, education level, years living in the LTC home, and family/care partner support. Most of the participants in this study were female. This finding is consistent with the gender distribution in LTC, as 90% of paid staff in LTC are women [52].

In an ideal situation, a randomized controlled trial would have been conducted, but given the constraints and challenges of COVID-19, this was an opportunity to explore a useful tool in a different format. In future research, studies could look to compare the usual PIECES format versus virtual PIECES. Family/care partners were provided with a one-page summary describing the virtual PIECES intervention. Despite receiving this information, some family/care partners were still unsure about the purpose of virtual PIECES. Future studies, especially those that include partnering with families and residents, should ensure that participants have full understanding of interventions and be provided with opportunities to ask questions. A potential strategy could be introducing the intervention at family and resident council meetings to offer opportunities for dialogue and clarification.

### Conclusion

This study entailed implementing a recognized clinical care planning tool (PIECES) in a virtual format to support the care of older adults experiencing responsive behaviors in LTC within the context of the COVID-19 pandemic. Findings suggest that virtual videoconferencing approaches to team-based care planning can be successfully implemented in LTC, and provide increased opportunities for engagement and inclusivity for LTC staff, family members, and older adults living in LTC. These findings are important because they indicate that family members can continue to be engaged in the care of older adults despite pandemic or outbreak situations, which may necessitate constraints on in-person participation. Moving forward, future research should explore how to effectively embed virtual care–planning processes more broadly into the policy and procedures of LTC homes, including understanding the required infrastructure, training, and supports needed to optimize inclusivity and equity. Important next steps could explore how virtual team–based care-planning approaches could potentially be used in remote locations to increase access to health care specialists such as geriatricians and nurses with geriatric specialization, and to foster engagement of family/care partners who may be unable to participate in person due to work or geographic limitations. Moreover, more research is needed to understand how virtual technology (eg, translation software) could increase engagement for older adults and family/care partners who are from linguistically and culturally diverse populations.

## Appendix

Multimedia Appendix 1 Consolidated Criteria for Reporting Qualitative Research (COREQ) checklist.

Multimedia Appendix 2 Sample focus group and interview questions.

## Abbreviations

COREQ: Consolidated Criteria for Reporting Qualitative Research
IT: information technology
LTC: long-term care
PSW: personal support worker
RN: registered nurse
RPN: registered practical nurse
