# Supplementary material for: Nurse-Led Virtual Delivery of PIECES in Canadian Long-Term Care Homes to Support the Care of Older Adults Experiencing Responsive Behaviors During COVID-19: Qualitative Descriptive Study
Source: JMIR Nurs. 2022 Dec 13;5(1):e42731. doi: 10.2196/42731 (PMC9762137; doi:10.2196/42731)
Supplement: Multimedia Appendix 2 [file nursing_v5i1e42731_app2.docx]

**Multimedia Appendix 2**

**Sample Focus Group and Interview Questions**

PIECES-Trained LTC Staff

- Tell me about a recent resident with behavioural expressions of concern.
  - What happened? What did you do?
  - Why? (Why did you do _____?)
  - What triggered this/why did it occur?
  - What seemed to work?
  - What didn’t work?
  - What could have worked better to address the care of the resident?
- How would you determine your approach for a resident with behavioural expressions of concern?
  - What factors play into your assessment/care planning?
  - Is your approach similar or different to what you see in your colleagues’ approach?
  - What role does the family play in your approach?
  - Have there been changes in practices or policies within your LTC home which have influenced your approach to care for residents with behavioural expressions?
  - How has your approach to providing care for residents with behavioural expressions evolved with your knowledge and experience?
- Tell me about a time when you utilized the PIECES approach with virtual team collaboration for a resident with behavioural expressions of concern.
  - What worked well? What were your successes?
  - What were the challenges?
  - How did you address those challenges?
  - What might help in the future?
  - What resources or supports would be helpful to you to overcome these challenges?
- How has the use of the PIECES approach with virtual team collaboration (video conferencing) influenced your practice?
  - Engagement of family members? Describe how you used the virtual technology to engage family. How does involving family members in the care of residents fit into your daily work experience?
  - Collaboration of team members? How does the virtual team collaboration aspect of PIECES fit into your daily work experience?
  - Collaborative care plan?

PIECES Mentors

- Can you describe the mentoring process for me?
- How did you facilitate “piloting” the process with the internal PIECES teams at each home prior to full-scale implementation?
- What feedback have you had about the mentoring process?
- How complicated is the implementation process of the PIECES approach with virtual care conferences?
  - Please consider the following aspects of the intervention: duration, scope, intricacy and number of steps involved and whether the intervention reflects a clear departure from previous practices.
- What feedback have you received on the quality of supporting materials, packaging, online resources, templates etc.?

Family/Care Partners

- Has your loved one experienced a change in behaviour expression (eg. agitation, wandering, or depression) that may have been upsetting or frustrating? Can you tell me about that time?
- What happened when the team from [LTC home] met with you and your family member for a care conference?
  - How did this care conference help you?
  - Was there a need for this PIECES discussion? (Why or why not?)
  - How do these PIECES conversations compare to other alternatives that you know about?
  - Tell me about your involvement with the virtual care conferences?
  - How do you feel about communicating virtually in meetings about care?
  - Do you feel your role was valued in these care conversations?
- What types of challenges have you faced when meeting virtually?
  - What happens to figure out problems?
  - Are you provided assistance to resolve any problems?
  - How are you encouraged to overcome these challenges?
  - What kinds of information and materials about PIECES have been made available to you?
  - Who do you ask if you have questions about PIECES or its implementation? How available are these individuals?
  - What kinds of changes or alterations do you think would help to make using the PIECES approach more effective at your LTC home?
- Tell me about a recent virtual care conference? What was your experience with the PIECES approach?
  - What happened?
  - How do the recommendations about care get done?
  - How does the team check in with you about whether the changes in care are going well or not so well?

Older Adult Research Partners

- Can you tell me about your experience working on this research project?
- What motivated you to want to be involved in the research?
- Is there anything from here on out that you would like to see changed or done differently with PIECES?
- Would you say as a result of your participation in this research project, you’re better informed about the PIECES approach and care planning?

RPN Champions

- Can you describe what you have done (or plan to do) to get a plan in place to implement the PIECES approach in this study?
  - What is your role in the planning process?
  - Who else is involved in the planning process? What are their roles?
- Can you describe the plan for implementing the intervention?
- How do you feel about the virtual PIECES care conferences being used in your setting?
  - Why is the intervention being implemented in your LTC home?
  - How do you feel about the plan to implement the intervention in your setting?
  - How do you think the program is going?
- What is your motivation for wanting to ensure the implementation is successful?
  - What kinds of incentives are there to help ensure the success of the implementation?
- What barriers does your organization face to implementing the virtual PIECES care conferences?
  - What kinds of feedback have you received thus far on the intervention? Can you describe a specific story?
